# Identification and population genetic analyses of copy number variations in six domestic goat breeds and Bezoar ibexes using next-generation sequencing

**DOI:** 10.1186/s12864-020-07267-6

**Published:** 2020-11-27

**Authors:** Jiazhong Guo, Jie Zhong, George E. Liu, Liu Yang, Li Li, Guangling Chen, Tianzeng Song, Hongping Zhang

**Affiliations:** 1 College of Animal Science and Technology, Sichuan Agricultural University, Chengdu, 611130 China; 2 Animal Genomics and Improvement Laboratory, BARC, Agricultural Research Service, USDA, Beltsville, MD 20705 USA; 3 Sichuan Technical Exchange Center, Science & Technology Department of Sichuan Province, Chengdu, 610016 China; 4 Institute of Animal Science, Tibet Academy of Agricultural and Animal Husbandry Sciences, Lhasa, 850009 China

**Keywords:** Goat, Copy number variations, Short-read sequencing, Comparative genomics

## Abstract

**Background:**

Copy number variations (CNVs) are a major form of genetic variations and are involved in animal domestication and genetic adaptation to local environments. We investigated CNVs in the domestic goat (*Capra hircus*) using Illumina short-read sequencing data, by comparing our lab data for 38 goats from three Chinese breeds (Chengdu Brown, Jintang Black, and Tibetan Cashmere) to public data for 26 individuals from three other breeds (two Moroccan and one Chinese) and 21samples from Bezoar ibexes.

**Results:**

We obtained a total of 2394 CNV regions (CNVRs) by merging 208,649 high-confidence CNVs, which spanned ~ 267 Mb of total length and accounted for 10.80% of the goat autosomal genome. Functional analyses showed that 2322 genes overlapping with the CNVRs were significantly enriched in 57 functional GO terms and KEGG pathways, most related to the nervous system, metabolic process, and reproduction system. Clustering patterns of all 85 samples generated separately from duplications and deletions were generally consistent with the results from SNPs, agreeing with the geographical origins of these goats. Based on genome-wide *F*_ST_ at each CNV locus, some genes overlapping with the highly divergent CNVs between domestic and wild goats were mainly enriched for several immunity-related pathways, whereas the genes overlapping with the highly differentiated CNVs between highland and lowland goats were mainly related to vitamin and lipid metabolism. Remarkably, a 507-bp deletion at ~ 14 kb downstream of *FGF5* on chromosome 6 showed highly divergent (*F*_ST_ = 0.973) between the highland and lowland goats. Together with an enhancer activity of this sequence shown previously, the function of this duplication in regulating fiber growth deserved to be further investigated in detail.

**Conclusion:**

We generated a comprehensive map of CNVs in goats. Many genetically differentiated CNVs among various goat populations might be associated with the population characteristics of domestic goat breeds.

**Supplementary Information:**

The online version contains supplementary material available at 10.1186/s12864-020-07267-6.

## Background

As a major class of structural variations (SVs) that complement to single nucleotide variations (SNVs), copy number variations (CNVs) refer to duplications, deletions, and insertions of DNA sequences ≥50 bp in size between individuals within a species [1]. A growing body of work demonstrates important effects of CNVs on phenotypic variations of Mendelian and quantitative traits in domestic animals by various molecular mechanisms, such as gene dosage, gene disruption, and gene fusion. For example, a previous work demonstrated that a massive amplification of a duplicated sequence in intron 1 of *SOX5* causes Pea-comb in chickens [2]. An inverted duplication and junction of two distinct regions of the chicken genome disrupt long-range cis-regulatory elements of *EDN3* and thereby result in the Silkie phenotype (i.e., dermal hyperpigmentation) [3]. A genome-wide association study and whole-genome sequencing identified a significant correlation between the degree of white spotting and a 1 Mb copy number region harboring *EDNRA* in Boer goats [4]. A 12.1-kb large deletion at the *HMGA2* locus results in small size in dwarf rabbits [5]. A 660-Kb deletion harboring four genes affects fertility and milk production traits antagonistically in Nordic red cattle [6], illustrating the occurrence of balancing selection in livestock. However, there are few studies concerning genome-wide characteristics of CNVs in goats so far [7, 8].

Similar to SNPs, the population genetic properties of CNVs could reflect genomic changes driven by evolutionary factors (e.g., natural/artificial selection, demographic history, and genetic drift) [9–13]. A well-known example of positive selection is that the copy number of the salivary amylase (*AMY1*) gene highly differs between the human populations with high-starch diets and those with traditionally low-starch diets [9]. Similarly, a genomic region harboring *AMY2B* was identified as a signature of dog domestication and modern dogs show high *AMY2B* copy numbers compared to wolves [14], demonstrating important roles of CNVs during animal domestication. A recent study of genome-wide CNVs in eight cattle breeds detected many lineage-differentiated CNVs that might be due to the selection for different traits (e.g., parasite resistance, body size, and fertility) [15]. In domestic goats, a genomic comparison with Bezoar showed that many copy number variable genes were associated with coat color, immune response, and production traits, which provided a clue for goat domestication [16]. Based on the ADAPTmap data, Liu et al. reported a population differentiation in CNV across different geographical areas around the world, suggesting the population history of different domestic goat breeds [7]. However, it is still limited with respect to the population genetic analyses using large-scale CNV data in domestic goats.

Compared to SNP chips and CGH arrays, next-generation sequencing (NGS) or short-read sequencing technologies have been matured rapidly in the past years, allowing us to identify and genotype CNVs more comprehensively and precisely at the genome-wide level [17, 18]. In this study, we aimed to investigate the genetic diversity, population structure, and population differentiation in domestic goats, based on CNVs detected using the whole-genome sequencing data from our previous work as well as the public data.

## Results

### CNVs distribute ubiquitously in the goat genome

**Fig. 1:**
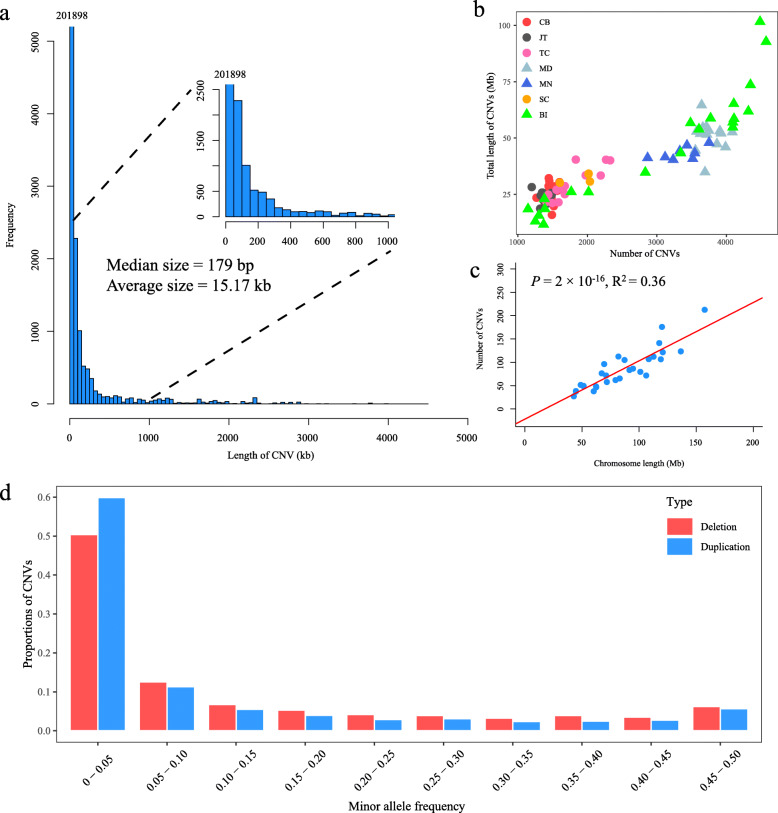
Genome-wide characteristics of CNVs in the goat genome. **a** A distribution histogram of the CNV length. **b** Total lengths and total numbers of CNVs identified in each goat samples. **c** The correlation between the number of CNVs on each chromosome and the chromosome length. **d** Frequency spectrum of all the deletions and duplications in seven goat populations as a whole group. Loci with the missing genotype rate > 10% were excluded

Based on the short-read sequencing data for 85 goats with the coverage depth of 5.25× ~ 15.90× (Additional file 1), we identified a total of 208,649 CNVs, including 17,876 duplications and 190,773 deletions across all autosomes (Additional file 2). As shown in Fig. 1a, the size of all the CNVs showed an L-shaped distribution (the median size = 179 bp, the average size = 15.17 kb), and the CNVs with ≤50 kb in size accounted for 96.76% (201,898) of the total CNV number. At the individual level (Fig. 1b), we discovered, on average, 2455 CNVs per goat genome with a range from 1145 to 4572, and the identified CNVs in each animal covered to 0.47 to 4.12% of the goat autosomal genome (2.47 Gb). It is noted that the number of identified CNVs in each goat appeared to increase with sequence depth in general. Based on a linear regression analysis, there was a strong positive relationship (*P* < 2.0 × 10^− 16^, F-test) between the number of CNV events on each chromosome and the chromosome length (Fig. 1c). We also constructed site frequency spectra of duplications and deletions in seven goat populations as a whole (Fig. 1d). The loci with MAF ≤ 0.05 accounted for 59.88 and 50.38% of the total duplication and deletion loci, respectively, suggesting that many CNV loci were skewed towards rare CNVs.

### The validation of CNVs by PCR and a second independent run of deep sequencing

We performed quantitative real-time PCR (qPCR) to test the accuracy for the CNVs detected by next-generation sequencing. The PCR results for all the seven deletions (~ 14 kb downstream of *FGF5*, ~ 470 kb downstream of *GABRB3*, *FOXO3*, *IGLON5*, *PRSS22*, *RARB*, and *KCNU1*) and one duplication harboring the complete *ASIP* and *AHCY* genes were all in agreement with our NGS prediction (Additional file 3).

To validate the detection accuracy of CNVs, we also carried out a second independent run of whole-genome deep sequencing for nine sampled goats (i.e., three individuals in each of the CB, JT, and TC goat breeds). After alignment against the reference genome (ARS1), the second runs of whole-genome sequencing data of the nine goats showed coverage depth of 25× at least (Additional file 1). The concordance rates for nine samples ranged from 68.23% (duplications in JT9 using Pindel) to 97.56% (duplications in CB10 using DELLY), suggesting the reliabilities of the CNVs identified above (Additional file 4).

### CNVRs were enriched with genes mainly related to nervous system and immune system

By merging overlapping CNVs across all the 85 goat samples, we finally obtained 2394 CNVRs (1722 deletions, 148 duplications, and 524 complex CNVRs) in all seven goat populations as a whole (Additional file 5). These CNVRs covered 266.71 Mb (10.80%) of the goat autosomal genome (2.47 Gb), and the median and average size of CNVRs was 2415 bp and 111.41 kb, respectively. The total number and length of the CNVRs identified in each population were provided in Additional file 5.

**Fig. 2:**
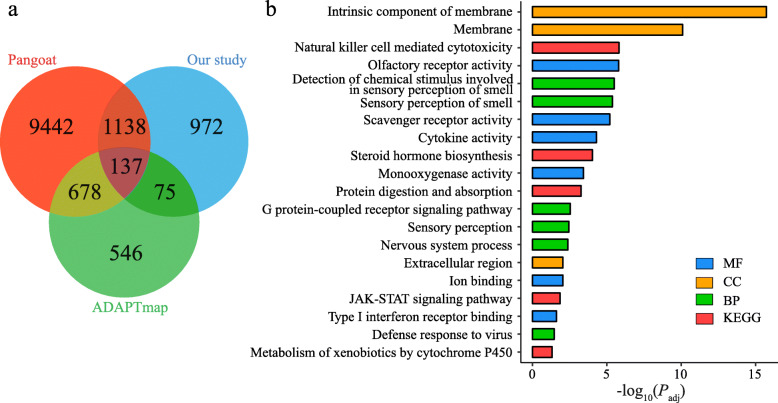
Summary of the copy number variable genes identified in the seven goat populations. **a** Comparison of the identified copy number variable genes with two other large-scale datasets. **b** Twenty representative functional enrichment GO and KEGG terms for CNV-overlapped genes

From genome annotation (ARS1), we obtained a total of 2322 genes (gene symbols) overlapping with the CNVRs (hereafter referred to as CNV genes) in the seven goat populations (Fig. 2a and Additional file 5). The functional enrichment analysis showed that these CNV genes were significantly enriched in 57 GO terms and KEGG pathways (*P*_adj_ < 0.05), which were mainly related to sensory perception of smell system (e.g., olfactory receptor activity, detection of chemical stimulus involved in sensory perception of smell, and sensory perception of smell), nervous system (i.e., sensory perception, nervous system process) and immune system (e.g., defense response to virus, scavenger receptor activity, and natural killer cell mediated cytotoxicity) (Fig. 2b and Additional file 6). Particularly, the term of olfactory receptor activity included 86 OR genes (Ensemble ID) associated with CNVRs (Additional file 5).

To further examine the credibility of the CNVRs detected in this study, we compared the CNV genes with those in the Goat Pan-genome database (hereafter referred to as Pangoat for simplicity) and a previous study [7] that used SNP data from the ADAPTmap project (see details in Methods). As shown in Fig. 2a, a total of 137 CNV genes (e.g., *ADAMTS20*, *ASIP*, *DGAT1*, and *AP2A2*) were shared among three datasets, suggesting that these CNVRs were common in a variety of goat populations. Furthermore, 54.91% of the total 2322 CNV genes (e.g., *ADCY5*, *CCND1*, *SOX13*, *SPATA5*, and *TLR6*) were present in the Goat Pan-genome database, whereas only 9.13% of the total CNV genes (e.g., *EXOSC4, IRF7*, and *MAPK15*) were shared with the CNV genes identified from the ADAPTmap project.

Considering the importance of coat color in goats, we attempted to examine whether our CNVRs overlap with pigmentation genes. Interestingly, we found a large duplication with 154,674 bp in length that encompasses the complete *ASIP* and *AHCY* genes and a partial sequence of *ITCH* at 63,226,821–63,381,495 bp on chromosome 13. This duplication was detected not only in white breeds (SC) but also in black goats (JT). Based on genotyping information from the NGS data, all the four SC goats (white coat color) were heterozygous for the two-copy *ASIP* allele (hereafter referred to as CN2-ASIP), while the CN2-ASIP allele showed a frequency of 11.11% in JT.

### The population structure analysis of goat populations

**Fig. 3:**
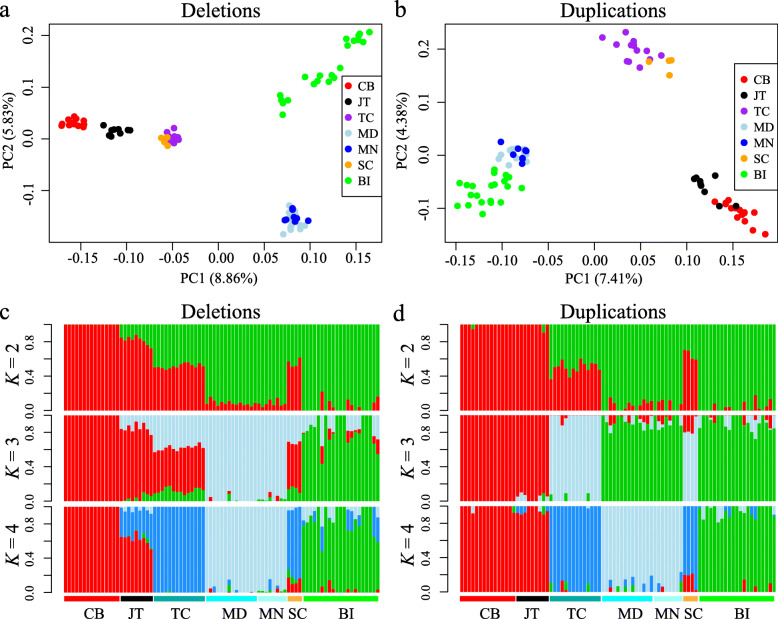
Population genetics analyses of the seven goat populations based on genome-wide deletion and duplication genotypes. **a** PCA of all 85 sampled goats based on genome-wide deletion genotypes. **b** PCA of all 85 sampled goats based on genome-wide duplication genotypes. **c** Population genetic structure of all 85 sampled goats inferred from genome-wide deletion genotypes. **d** Population genetic structure of all 85 sampled goats inferred from genome-wide duplication genotypes

The genome-wide deletion or duplication genotyping information allowed us to examine the population structure in seven goat populations. In terms of deletions, the PCA analysis using deletions (variance explained = 8.86 and 5.83% for PC1 and PC2, respectively) and duplications (variance explained = 7.41 and 4.38% for PC1 and PC2, respectively) showed that the 85 goat samples mainly clustered into three large groups: Chinese breeds (i.e., CB, JT, TC, and SC), African breeds (i.e., MD and MN), and Bezoar ibexes (Fig. 3a). Based on duplications (Fig. 3b), the both Moroccan breeds and Bezoar ibexes clustered closely, whereas four Chinese breeds can be divided into two large groups: TC and SC (i.e., Cashmere goats), CB and JT (both breeds were from the Sichuan Basin). When the genetic admixture analysis was conducted by assuming different numbers of ancestral populations (K = 2 ~ 7), the results generated from deletions showed that K = 3 was the most plausible number of genetically distinct groups (Fig. 3c). In this scenario, the ancestors of the 85 sampled goats can be resolved three populations: a wild goat population, a Moroccan goat population, and a Chinese goat population. According to duplications, all the sampled goats were thought to be most likely (K = 2) from two ancestral populations: domestic population and a wild population (Fig. 3d).

### The differentiated CNVs between domestic goats and Bezoar ibexes were mainly related to immune system

**Fig. 4:**
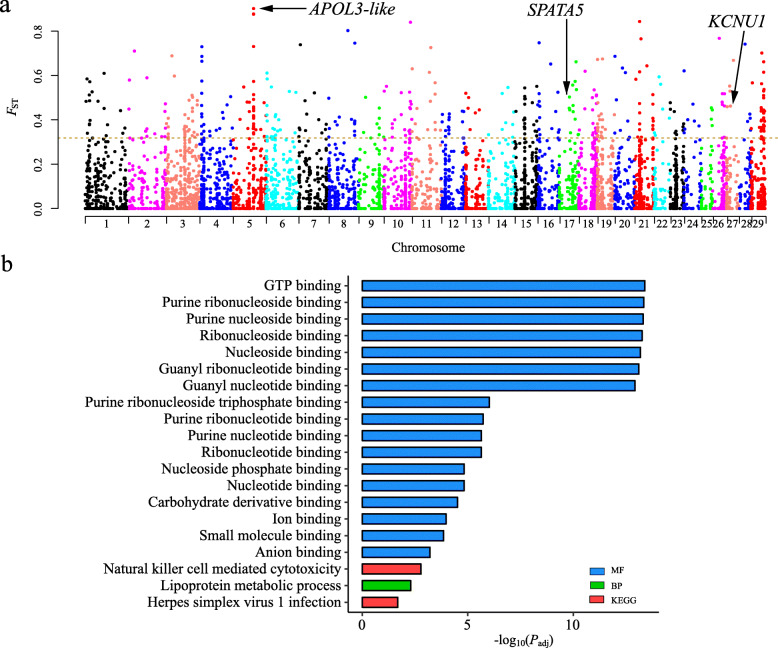
Comparative genomic analysis between domestic goats and Bezoar ibexes using population fixation index (*F*_ST_). **a** Manhattan plot of genome-wide *F*_ST_ on each CNV locus between Bezoar ibexes and two domestic goat populations (MD and MN) as a whole group. The horizontal dashed line indicates the 95 percentile of all the *F*_ST_ values. **b** Top twenty enriched GO terms and KEGG pathways for the genes overlapping with highly differentiated CNVs between Bezoar ibexes and two domestic goat populations

To find the CNVs that were genetically differentiated between domestic goats (i.e., MD and MN) and Bezoar ibexes, we carried out a genome-wide scan by estimating the genome-wide fixation index (i.e., *F*_ST_) at each CNV locus. To reduce the impact of the differences in sequencing depths, here we only selected 13 Bezoar ibexes that had similar sequencing depths with the goats from MD and MN breeds. The genome-wide average *F*_ST_ was 0.068 (median *F*_ST_ = 0.009) across a total of 8792 CNV loci (6544 deletions and 2248 duplications). Based on the 5% right tail (i.e., *F*_ST_ ≥ 0.318) of the empirical *F*_ST_ distribution, 450 outlier loci that overlapped with 194 genes (Ensemble ID) were thought to be highly divergent between domestic goats and Bezoar ibex (Fig. 4a and Additional file 7). The functional analysis showed that the genes overlapping with the differentiated CNVs were significantly enriched (*P*_adj_ < 0.05) in the functional terms of immune system (e.g., natural killer cell mediated cytotoxicity, Herpes simplex virus 1 infection, and type I interferon receptor binding), metabolism (e.g., lipoprotein metabolic process) and digestion (carbohydrate derivative binding) (Fig. 4b and Additional file 8). The genome-wide distribution of global *F*_ST_ revealed the highest differentiated hit (*F*_ST_ = 0.903) was a 26,120-bp duplication (chromosome 5: 73,049,912–73,076,032 bp) that overlapped with the gene *APOL3-like* (Fig. 4a and Additional file 7). Additionally, we identified a 55-bp deletion (chromosome 17: 36,494,821–36,494,876 bp) in *SPATA5* and an 83-bp deletion (chromosome 27: 13,435,433–13,435,516 bp) in *KCNU1*.

### The differentiated CNVs between highland goats and lowland goats included a 507-bp deletion at the ~ 14 kb downstream of *FGF5*

**Fig. 5:**
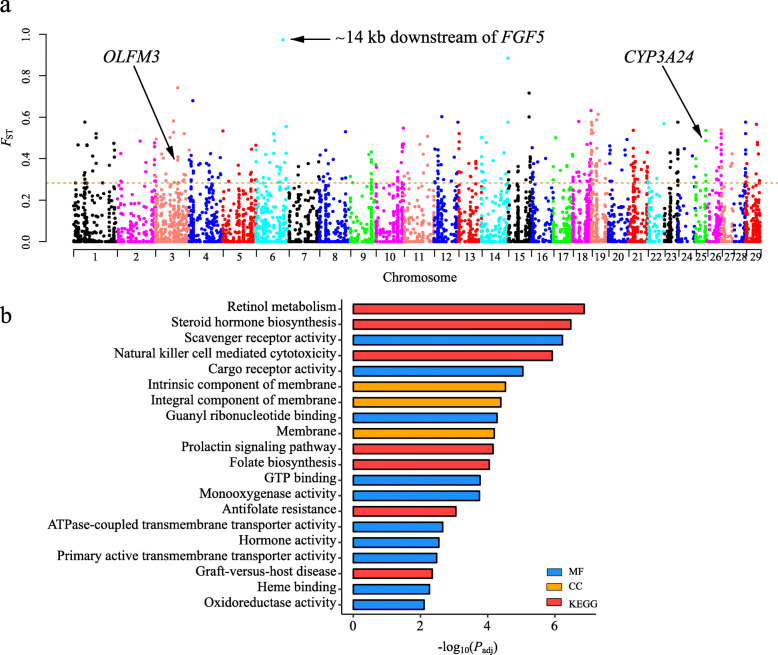
Comparative genomic analysis between highland breeds (TC) and lowland breeds using population fixation index (*F*_ST_). **a** Manhattan plot of genome-wide *F*_ST_ on each CNV locus between TC and two domestic goat populations (i.e., CB and JT) as a whole group. **b** Top twenty enriched GO terms and KEGG pathways for the genes overlapping with highly differentiated CNVs between TC and two lowland populations

To identify the CNV loci that underlie genetic adaptation to high-altitude, we conducted genome-wide estimates of single-maker *F*_ST_ between a highland breed (i.e., TC) and two lowland breeds (i.e., CB and JT) as a whole control group. The genome-wide average *F*_ST_ was 0.064 (median *F*_ST_ = 0.012) across a total of 7896 CNV loci (5873 deletions and 2023 duplications). Based on the 5% right tail (i.e., *F*_ST_ ≥ 0.282) of the empirical *F*_ST_ distribution, 398 outlier loci that overlapped with 317 genes (Ensemble ID) were thought to be highly divergent between TC and the lowland breeds (Fig. 5a and Additional file 8). The functional terms significantly enriched for the differentiated CNV genes (*P*_adj_ < 0.05) were mainly related to vitamin and lipid metabolism (e.g., retinol metabolism, folate biosynthesis, and steroid hormone biosynthesis), immune system (e.g., scavenger receptor activity and natural killer cell mediated cytotoxicity), and reproduction (i.e., prolactin signaling pathway) (Fig. 5b and Additional file 9). For example, the CNV genes including eight cytochrome-P450 (*CYP450*) genes (e.g., *CYP2S1*, *CYP2C31*, and *CYP3A24*) were significantly enriched in monooxygenase activity. Additionally, six olfactory-related genes (e.g., *OLFM3*, *OR6C1,* and *OR6C3*) overlapped with the differentiated CNVs.

**Fig. 6:**
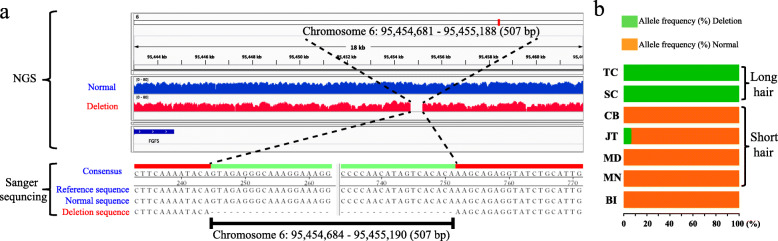
Characterization of a 507-bp deletion at ~ 14 kb downstream of *FGF5* on chromosome 6. **a** The screen capture of aligned short sequencing reads featuring a 507-bp deletion at ~ 14 kb downstream of *FGF5* on chromosome 6 using IGV. **b** The allele frequency of this deletion in six domestic goat populations and Bezoar ibexes

Among all the analyzed CNV loci, the highest differentiated hit (*F*_ST_ = 0.973) was a 507-bp deletion on chromosome 6 that located at 95,454,681–95,455,188 bp and ~ 14 kb downstream of *FGF5* that is a major regulator affecting hair growth and development (Fig. 5a and Fig. 6a). Interestingly, an almost same deletion (a 504-bp deletion at 95,454,685–95,455,188 bp of chromosome 6) was recently identified in Chinese Cashmere goats with a high frequency [19]. We also examined the frequency patterns of this deletion among the analyzed goat population in this study. Based on the NGS data, this deletion was fixed (frequency = 100%) in TC (*n* = 14) and SC (*n* = 4), whereas the goat breeds with short hair do not carry this deletion except for one goat in JT (Fig. 6b).

## Discussion

### The characteristics of CNVs in the goat genome

In this study, we used the whole genome short-read sequencing data of 85 individuals from six domestic goat populations and Bezoar ibexes to generate a detailed CNV map in the goat genome. In total, 2394 CNVRs were obtained by merging 208,649 high-confidence CNVs across all the goat samples, covering 10.80% of the goat autosomal genome. This is in line with the total coverage of CNVs in humans (12, 13%) [20, 21], cattle (6 and 8.9%) [22, 23], sheep (6.9%) [24], and yak (6.2%) [25]. For example, similar to the distribution in the cattle genome [22], the number of CNV events on each chromosome increased with the chromosome length. Compared to previous genome-wide studies [7, 8], we detected many more CNVs in the goat genome, which can be attributed to the differences in genetic data types (e.g., SNP chips and whole-genome sequencing) and the sampled populations. Notably, high amounts of CNVs with small sizes (~ 100 kb or less) resulted in an L-shaped distribution of the size of all CNVs identified here, supported by the results in other livestock breeds (e.g., cattle [22], pigs [26], and sheep [24]) and humans [27, 28]. Theoretically, CNVs in larger sizes (e.g., > 100 kb) change the imbalances or dosage effects of more genes in comparisons with the CNVs in smaller sizes [29], which results in that large CNVs are generally more deleterious at the phenotypic level and are thus relatively rare in a population.

### Comparisons with previous large-scale detections of CNVRs in goats

Many genes (e.g., *ADAMTS20*, *ASIP*, *DGAT1*, and *AP2A2*) overlapping with the CNVs identified in this work are also present in the Pangoat database, supporting our results and these CNVs are likely common in domestic goats (Fig. 2a). However, there were fewer shared CNV genes (e.g., *ADAMTS20*, *ASIP*, and *DGAT1*) between our work and the result obtained from the ADAPTmap data [7], which can be explained by different sampled populations (i.e., the ADAPTmap data included few samples from East Asia) and suggested that many novel CNVRs were identified in our study. For example, different members of the olfactory gene family that is characterized by frequent duplications and losses in humans [30] and vertebrates (e.g., chimpanzees [20], mouse [21], and fishes [23]) were identified in these goat datasets and this work. The Pangoat database not only collected the latest goat reference genome (ARS1), but also used nine de novo assemblies from seven *Caprini* species (e.g., sheep, argali sheep, mouflon sheep, and ibex) to construct the CNVR dataset. It additionally included multiple whole genome resequencing data, and thereby result in a larger total number of CNVR than ours.

As mentioned above, several widely distributed CNVRs in domestic goat breeds deserved more attentions, because they overlapped with the genes (e.g., *ASIP*, *DGAT1*, *PRP1*, and *PRP6*) known for their biological functions in livestock. For example, we found that one 154-kb CNV harboring *ASIP* on chromosome 13 was present in a white coated breed (i.e., SC) and two non-white coat breeds (i.e., JT and TC). It is generally recognized that *ASIP* is a key gene regulating the production of pheomelanin and eumelanin in animals [25], and thereby SNPs or short indels within this gene were primarily associated with yellow/red and black/brown coat color in animals including horses [31], pigs [32], and sheep [33]. A 190-kb duplication harboring the *ASIP* and *AHCY* coding regions and the *ITCH* promoter sequence was determined as the casual variants for the dominant white coat color in sheep [34]. However, the duplication harboring *ASIP* was present in five goat breeds with different coat colors (e.g., white, red, and black) from Italy [35], which was consistent with our finding. Furthermore, the copy number of a genomic region containing *ENDRA* was remarkably correlated with decreasing pigmentation in Boer goats [4]. Considering all together, there was no clear association of CNV of the *ASIP* gene in this study with white coat color. Therefore, future analyses with a large sample size and additional computational pipelines will be required to obtain a definite conclusion.

The encoded protein of *DGAT1* catalyzes the conversion of diacylglycerol and fatty acyl CoA to triacylglycerol, and this gene has been identified as a major locus for milk production traits in Holstein [36]. Similar to the discovery (chromosome 14: 80,740,427–81,602,889 bp) using the ADAPTmap data, a CNVR (chromosome 14: 80,629,343–81,407,250 bp; Additional file 6) covering *DGAT1* was not only found in Moroccan breeds from Africa, but also was present in the Chinese goat breeds of this study. The other examples are described below, including prolactin (PRL), a key reproductive hormone, has the functions of regulating ovarian function, maintaining pregnant corpus luteum and promoting fetal development [37]. The proliferin-related protein (PRP), as a placental-specific hormone, is mainly involved in the PRL signaling pathway [38]. We also found *PRP1* and *PRP6* genes, whose duplications were recently reported to be associated with high-fecundity in Laoshan dairy goats [39].

### Population genetic analyses based on CNVs

In this study, the PCA and population admixture analyses, based on the genome-wide duplication and deletion genotypes, display that all goat samples cluster into different groups, generally consistent with the geographical origins of the goat samples and the results generated from SNPs in our previous work [40]. Our population structure analyses demonstrated that partitioning of diversity among modern Chinese goat populations, African goat populations, and Bezoar ibexes, which was in line with the findings from the ADAPTmap data that include 1023 goats from 50 breeds around the world [7]. It is noteworthy that the results obtained from deletions were more similar to those from SNPs, which was also observed in humans [27] and monkeys [41]. Considering the limited locus numbers in our study, this difference might suggest that more genetic sites would result in higher resolutions in the investigations of population relationships.

### Domestication-associated CNVs

During domestication mainly driven by artificial selection, livestock species have evolved different phenotypic and genetic characteristics as compared to their wild progenitors, including morphological, behavioral, and physiological traits [42]. In this study, the most differentiated CNV loci between domestic goats and BI overlapped with *APOL3-like*, which might be a paralog of *APOL3* and is adjacent to *APOL3* in the goat genome. CNVs at the *APOL3* locus have been reported in several cattle breeds [43, 44] and found to be associated with adult cattle stature [45]. Furthermore, some genes overlapping with the highly divergent CNVs between domestic and wild goats were enriched in the immune system, which was consistent with the reports that some immunity-related CNV genes evolved rapidly between domestic and wild goats (*Capra aegagrus*) [16], and between domestic pigs and wild boars [46]. These findings collectively suggested the biological processes underlying the domestication were similar in different livestock species.

Notably, some genes play roles in spermatogenesis and sperm capacitation. For example, SPATA5 is a spermatogenesis-associated factor and is expressed specifically in the spermatogonia and spermatocytes [47]. A large deletion harboring *SPATA5* was associated with multiple malformations and hearing loss in humans [48]. The *KCNU1* gene, also known as *SLO3*, is expressed only in mammalian testis and plays a vital role in male fertility [49]. The CNV overlapping with *KCNU1* was recently reported in pigs [50].

### Several interesting CNVs in Tibetan cashmere goats

Although the relevant issues have been extensively investigated using SNPs, there were a few reports on the selection signals that underlie genetic adaptations to high altitude in humans and animals based on CNVs [51–53]. In this study, some genes overlapping with the CNVs, which were highly differentiated between Tibetan goats and lowland goat breeds, deserved more attention because of their known functions. As a superfamily of 56 functional genes, the *CYP450* gene family is mainly involved in drug metabolism and bioactivation in humans [54, 55]. The characteristics of several *CYP450* gene copy number have been investigated in different human populations [54, 56, 57], particularly for *CYP2D6* [58–60]. Interestingly, some *CYP450* genes were found to be related to genetic adaptations for the high altitude in horses [61] and frogs [62], which can be explained by their function as monooxygenases. Based on an CGH array, CNVs harboring *CYP2C* were also identified in a highland sheep breed (i.e., Mongolian sheep) [63], which was consistent with our findings.

Cashmere-related traits (e.g., fiber length) shaped by artificial selection and adaptation to low temperatures are the most important economic traits in Cashmere goat populations on plateaus of Asia. It is widely recognized that *FGF5* is a key regulator for hair length in animals [64–66], and its disruption via the CRISPR/Cas9 system leads to longer fibers in genome-edited goats [67, 68] and sheep [69]. However, the natural causal mutations affecting cashmere-related traits in goats remain elusive. We recently identified one SNP (c.-253G > A) in the 5′-UTR of *FGF5* that could introduce a start codon during translation, and a large allele frequency difference at this site was observed between long hair and short hair goats and goats [40]. In this study, we discovered a 507-bp deletion downstream of *FGF5* fixed in Tibetan Cashmere goats (TC) population. Remarkably, a previous study revealed that an almost same deletion (95,454,685–95,455,188 bp on chromosome 6) was present in all Chinese Cashmere goats at a high frequency, and this deletion can function as a enhancer [19], which was validated by a recent study in Chinese Cashmere goats [70]. Therefore, we proposed that both SNP and CNV mutations could contribute to cashmere-related traits in goats by regulating the expression of *FGF5*. However, more functional experiments are needed to fully uncover their biological functions in detail.

## Conclusion

We obtained a comprehensive map of CNVs in goats. Many genetically differentiated CNVs among various goat populations might be associated with the population characteristics of domestic goat breeds. Taken together, our findings provide a better understanding of the evolutionary history of domestic goats, and represent a valuable molecular variation resource for future work on examining genetic mechanisms of phenotypic variations and genetic improvement in goats.

## Methods

### Whole-genome sequencing data analysis

The information of sampled goats and short read alignment against the goat reference genome (ARS1) have been described in our previous work [40] but are included here for completeness. In brief, whole-genome sequencing data of four Chinese breeds (Chengdu Brown (CB, *n* = 15), Jintang Black (JT, *n* = 9), Tibetan Cashmere (TC, *n* = 14), and Shaanbei Cashmere (SC, *n* = 4)), two Moroccan breeds (Draa - MD, *n* = 14 and Northern - MN, *n* = 8), and 21 Bezoar ibexes were analyzed in this study. The goat samples from the CB, JT, and TC populations were collected by ourselves and were from a breeding farm in Dayi County, a breeding farm in Jintang County, and Coqen County in Tibet, respectively, and the animals were released after the sample collection. In contrast, the whole-genome sequencing data of the goat samples from three other domestic goat breeds (i.e., SC, MD, and MN) and Bezoar ibexes were downloaded from NCBI. According to the alignment against the goat reference genome (ARS1) using BWA (v0.7.12) [71], the coverage depth of whole genome sequencing data of 85 goats varied between 5.25× and 15.90× (Additional file 1). To improve accuracy of the identification of CNVs, duplicated reads were removed from raw alignments with the Picard software (v2.10.6) (http://broadinstitute.github.io/picard/), followed by local realignment around existing indels with GATK (v3.8–0) [72].

### CNV discovery and genotyping

Three software with different approaches (i.e., split-read, paired-end mapping, and read depth) were applied to detect duplications and deletions (Additional file 9). We firstly employed DELLY [73] and Pindel [74] with default parameters to detect raw CNVs in each goat genome, respectively. Raw CNVs were then filtered by applying the following thresholds: length > 50 bp and “PASS” loci (DELLY); length > 50 bp, variant allele frequency > 0.2, and supported read number ≥ 3 (Pindel). We also used CNVcaller [75] with default parameters to detect raw copy number variation regions (CNVRs) at the population level.

To further reduce redundancy and obtain high-confidence CNVs and CNVRs, we conducted the following quality control pipeline (Additional file 9): (1) After applying the command ‘intersect’ of BEDTools [76], we only retained the CNVs in each goat genome, if the CNVs detected with DELLY and Pindel overlap in the genomic coordinates and the overlapping sequence length accounted for ≥50% of a CNV size. We further redefined the length and location of these CNVs based on the overlapped sequence. (2) We then merged multiple adjacent CNVs into a CNVR across individuals within a population and at the meta-population level using the command ‘merge’ of BEDTools if the overlapping sequence was at least > 1 bp along their genomic coordinates [77], and discarded the CNVRs only including one CNV in each population or the meta-population. (3) The CNVRs derived from the above step and the CNVRs generated with CNVcaller were combined into a unique CNVR, if multiple CNVRs overlapped with ≥50% in size of the shortest CNVR. A raw CNVR was moved if it was only generated from one approach. Here, the CNVRs that exclusively include deletions or duplications were defined as deletion CNVRs and duplication CNVRs, respectively, whereas the CNVRs that comprised both deletions and duplications, we defined them as complex CNVRs.

Genome-wide deletion and duplication genotypes were obtained using DELLY [73]. First, all deletion/duplication sites across all samples were merged into a unified site list using the command ‘merge’ in DELLY. This merged CNV site list was then genotyped with the command ‘call’ in DELLY. All genotyped samples were merged into a single BCF using the command ‘merge’ in bcftools [78], and the BCF was converted to VCF with the command ‘view’ in bcftools. Finally, the genotypes of CNVs located in the unique CNVRs were extracted for downstream analyses with the command ‘intersect’ in BEDTools.

### The validation of CNVs by PCR and a second independent run of deep sequencing

PCR and quantitative PCR (qPCR) have been performed to validate the accuracy for CNVs detected by using next-generation sequencing. Seven deletions and one duplication were randomly selected for PCR validation using genomic DNA of the same samples. The primer information for PCR validation was provided in Additional file 10. The *MC1R* gene was used as the reference gene for qPCR experiments [8], and we calculated the relative copy number for each selected site using the 2^-ΔΔCT^ method.

We also conducted a second run of whole-genome deep sequencing data (> ~ 25×) for nine sampled goats (i.e., three individuals in each of the CB, JT, and TC goat breeds). The alignment of reads and identification of CNVs for these datasets were carried out using the workflow as described above. In addition, the alignments of animals were visualized with Integrative Genomics Viewer (IGV) [79] to confirm the existence of several CNV loci.

### CNV-based population genetic analyses

To explore the utility of CNVs in population genetic analyses, the genetic structure analysis was carried out with the ADMIXTURE [80] using genome-wide duplication and deletion genotypes in all sampled goats. Principal component analysis (PCA) implemented in GCTA [81] was also performed to examine population stratification at the individual level. Notably, we employed VCFtools [82] to remove the CNV loci with a minor allele frequency (MAF) lower than 0.05 and the loci with more than 10% missing genotypes at the meta-population level, before conducting the population structure analyses and other downstream population genetic analyses.

Comparative genome analyses of different goat groups were conducted by calculating genome-wide *F*_ST_ (i.e. Weir and Cockerham’s estimator [83]). To find the domestication-related CNVs, the two domestic goat breeds (i.e., MD and MN) were merged to be a whole group. We calculated genome-wide *F*_ST_ values at each CNV loci (MAF ≥ 0.05) between domestic goats and Bezoar ibexes. To detect the CNVs that are associated with adaptation to high-altitude, we also determined the pairwise *F*_ST_ at each CNV loci between the highland breed (TC) and the lowland control group of breeds (CB and JT). Based on the empirical *F*_ST_ distribution, we focused on the top 5% of CNV loci showing extremely high *F*_ST_ value, and tested whether these “outlier” loci were related to important traits in goats.

### Functional enrichment analysis of genes overlapping with CNVs

The genes that were located within or overlapped with CNVs were extracted based on genome coordinates. Functional enrichment analyses for these genes were then carried out using g:Profiler (https://biit.cs.ut.ee/gprofiler/gost) [84].

### Comparison of CNVR-overlapped genes with known large-scale CNV datasets

Our CNVRs were compared with two large-scale CNVR datasets: the CNVRs from the Goat Pan-genome database (http://animal.nwsuaf.edu.cn/code/index.php/panGoat) and the CNVRs from a previous study that used CaprineSNP50 genotyping data generated by the ADAPTmap project [7]. The CNVRs in the Pangoat database were obtained from the goat ARS1 genome assembly and other nine de novo assemblies from seven *Caprini* species (including sheep, argali sheep, mouflon sheep, wild goat, ibex, Barbary sheep, and blue sheep) as well as multiple resequencing data. Given the differences between platforms and pipelines, the genes overlapped with CNVRs in each dataset were compared.

## Supplementary Information

**Additional file 1: Table S1.** Summary of mapping statistics for 85 goat samples and nine high-depth resequenced goats included in this study.

**Additional file 2: Table S2.** List of CNVs identified in 85 goat genomes included in this study.

**Additional file 3: Figure S1.** The PCR results of validation experiments for eight CNV loci.

**Additional file 4: Figure S2.** Results of validation for the identification of CNVs using an independent run of deep sequencing data.

**Additional file 5: Table S3.** List of CNVRs and genes overlapping with CNVRs identified in this study.

**Additional file 6: Table S4.** Significantly enriched terms for the genes overlapped with 2394 CNVRs identified in seven goat populations.

**Additional file 7: Table S5.** List of highly differentiated CNVs between BI and domestic goats and between TC and lowland goats.

**Additional file 8: Table S6.** Significantly enriched terms for the differentiation CNV genes identified in this study.

**Additional file 9: Figure S3.** The pipeline used to identify CNVs and CNVRs. Each step was described in detail in the Methods section.

**Additional file 10: Table S7.** List of the primer information for the validation of CNVs by PCR and qPCR.

## Data Availability

The raw sequencing data of 85 animals for the identification of CNVs in this study are available from the NCBI SRA database (dataset numbers: PRJNA548681, PRJNA422206, PRJEB3136, PRJEB5900, and PRJEB3134). The deep sequencing data of nine goats for the validation in this study are also deposited at NCBI under accession number PRJNA642185.

## Abbreviations

BI: Bezoar ibex
CB: Chengdu Brown goats
CNV: Copy number variation
CNVR: CNV region
F_ST_: Fixation index
JT: Jintang Black goats
MAF: Minor allele frequency
MD: Moroccan Draa goats
MN: Moroccan Northern goats
NGS: Next-generations sequencing
SC: Shaanbei Cashmere goats
SNP: Single nucleotide polymorphism
SNV: Single nucleotide variation
TC: Tibetan Cashmere goats
