# Supplementary figures and images for: Identification and population genetic analyses of copy number variations in six domestic goat breeds and Bezoar ibexes using next-generation sequencing
Source: BMC Genomics. 2020 Nov 27;21:840. doi: 10.1186/s12864-020-07267-6 (PMC7694352; doi:10.1186/s12864-020-07267-6)

a

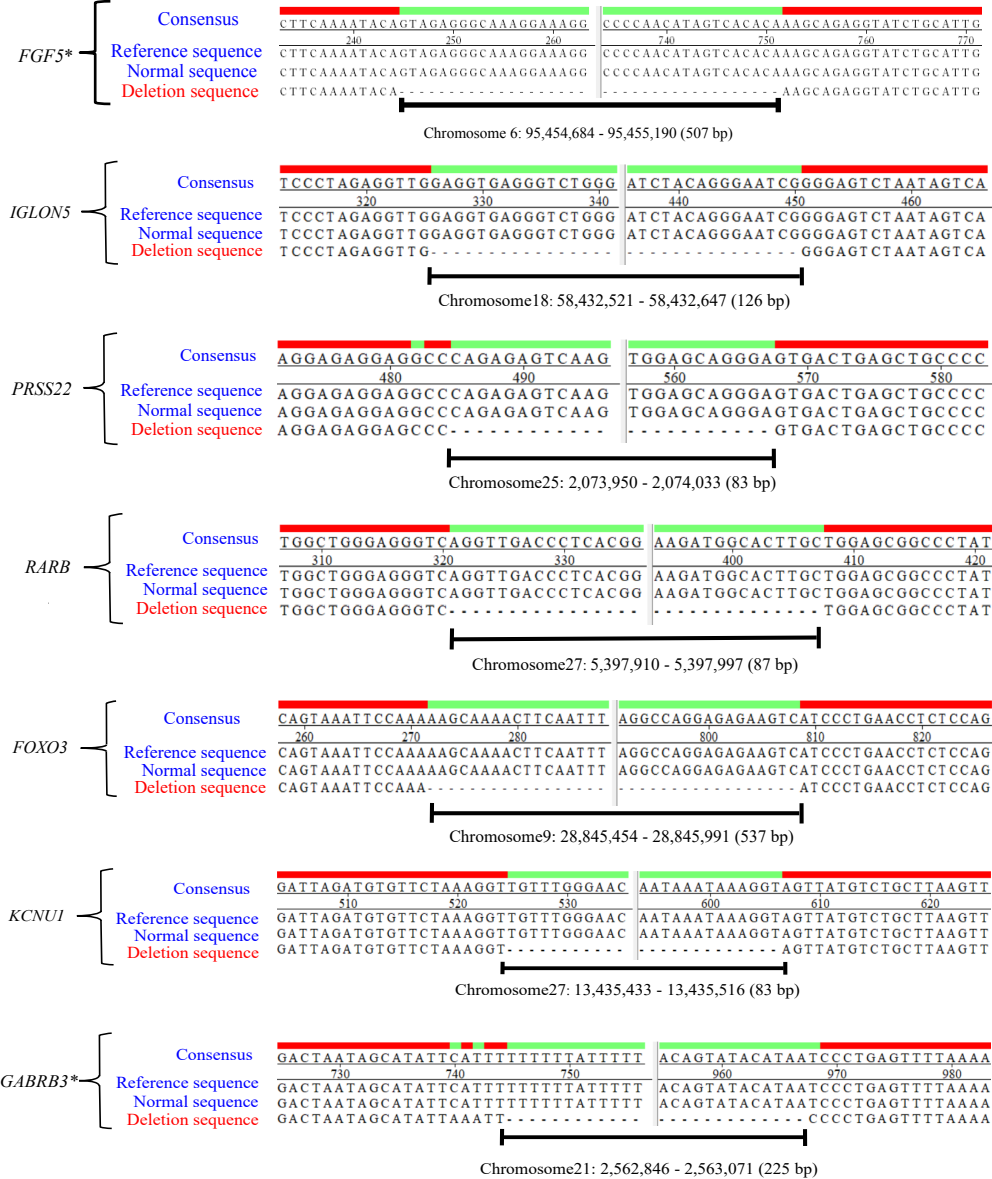

b

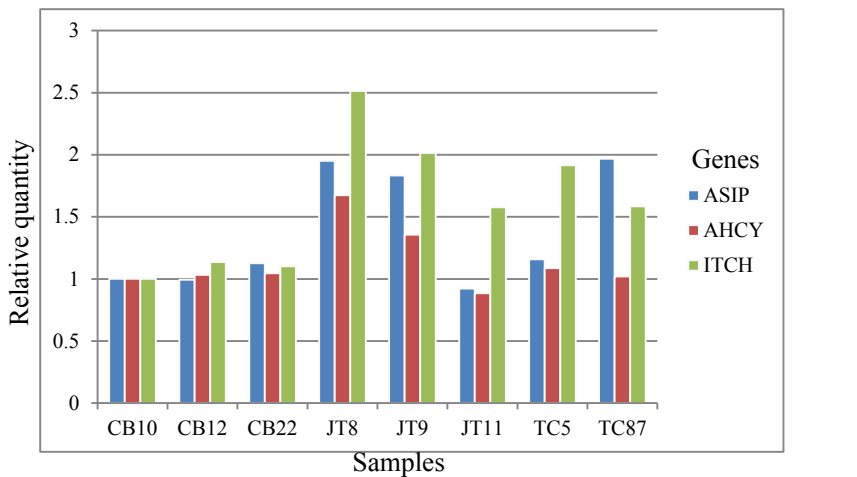

Supplement: Supplementary file 3 — Additional file 3: Figure S1. The PCR results of validation experiments for eight CNV loci. [file 12864_2020_7267_MOESM3_ESM.pdf]

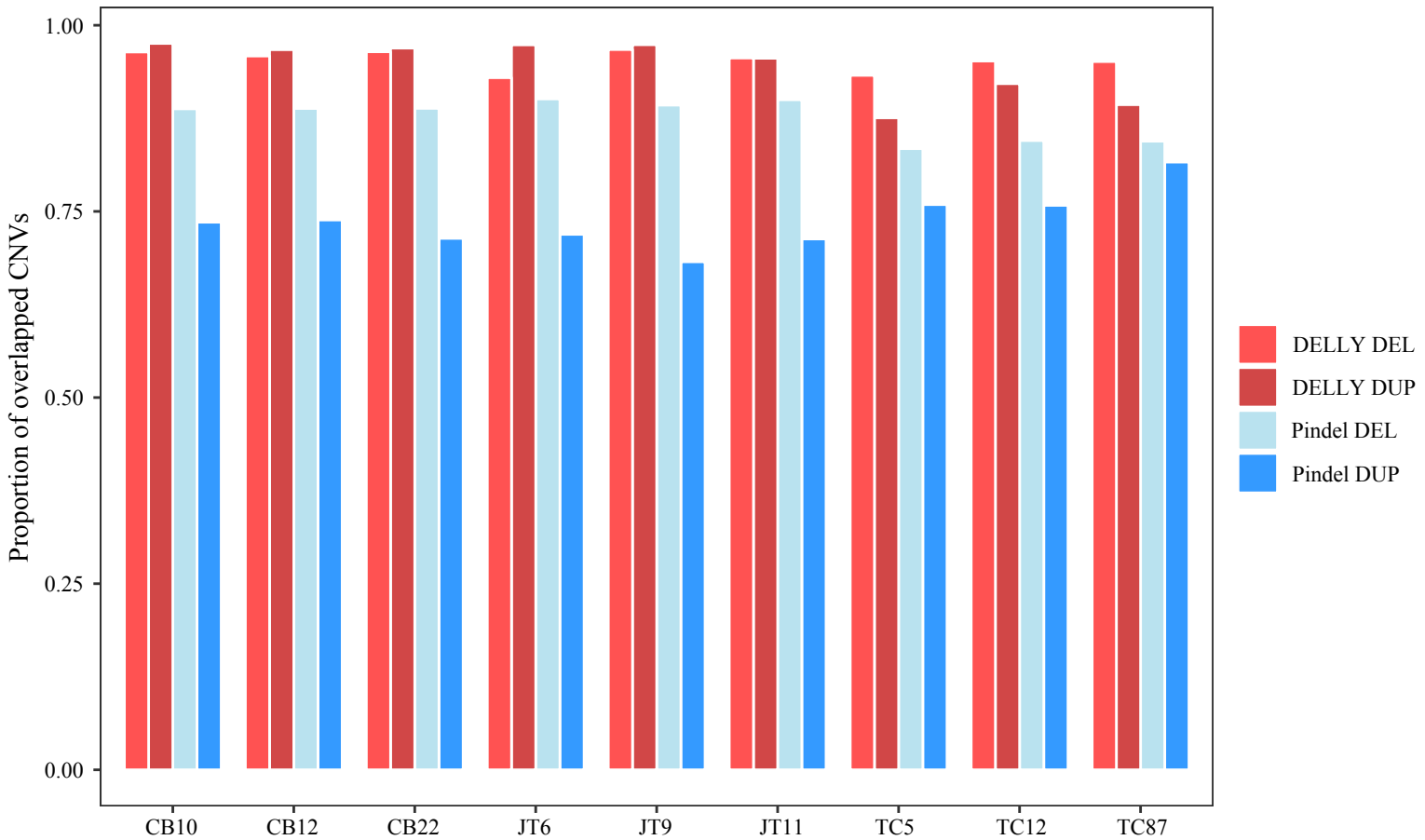

Supplement: Supplementary file 4 — Additional file 4: Figure S2. Results of validation for the identification of CNVs using an independent run of deep sequencing data. [file 12864_2020_7267_MOESM4_ESM.pdf]

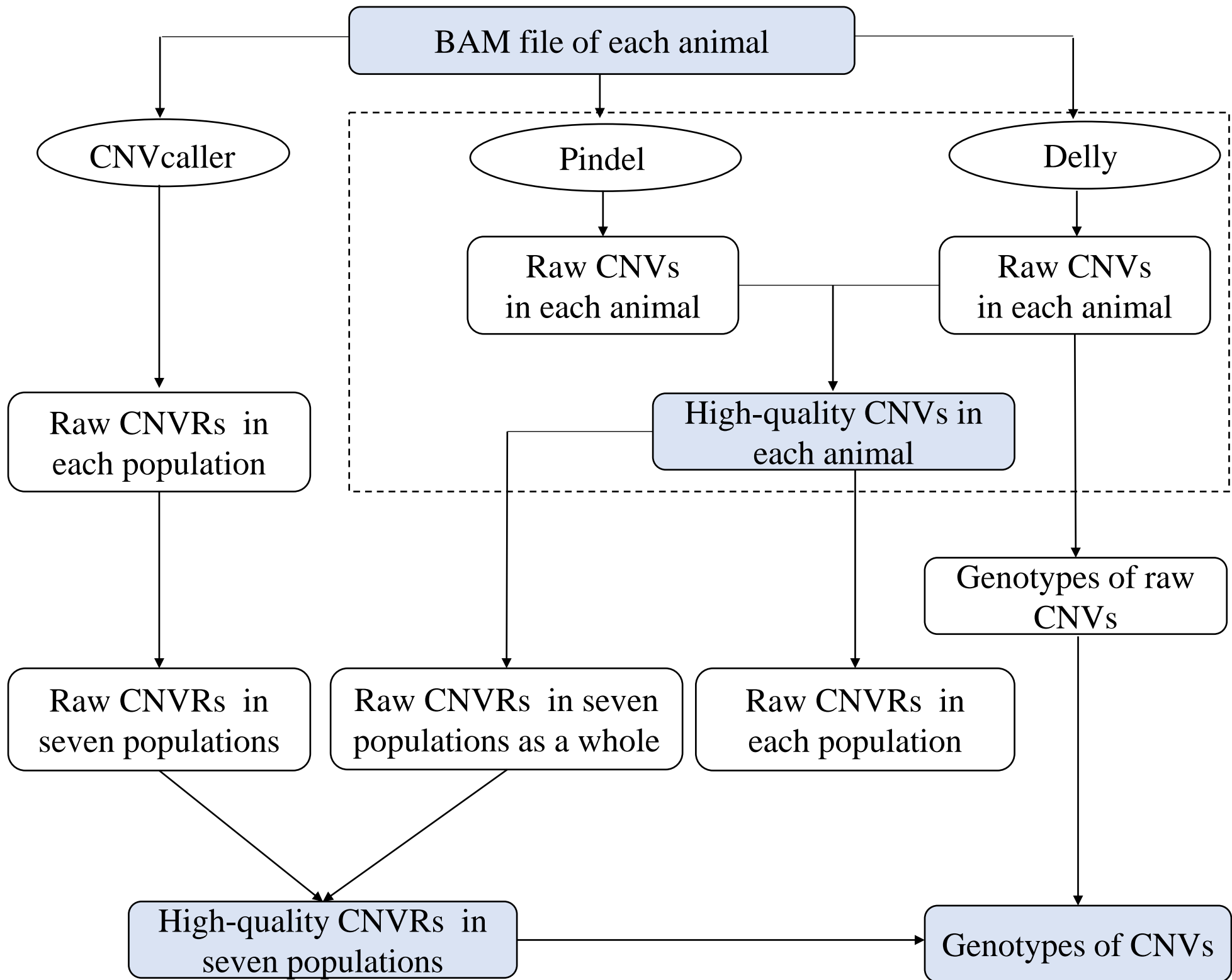

Supplement: Supplementary file 9 — Additional file 9: Figure S3. The pipeline used to identify CNVs and CNVRs. Each step was described in detail in the Methods section. [file 12864_2020_7267_MOESM9_ESM.pdf]
